# Culture-based characterization of gut microbiota in inflammatory bowel disease

**DOI:** 10.3389/fmicb.2025.1538620

**Published:** 2025-02-20

**Authors:** Hyunjoon Park, Soyoung Yeo, Taekyu Lee, Yumin Han, Chang Beom Ryu, Chul Sung Huh

**Affiliations:** ^1^Research Institute of Eco-friendly Livestock Science, Institute of Green-Bio Science and Technology, Seoul National University, Pyeongchang, Republic of Korea; ^2^Department of Agricultural Biotechnology, College of Agriculture and Life Sciences, Seoul National University, Seoul, Republic of Korea; ^3^Department of Internal Medicine, Digestive Disease Center and Research Institute, Soon Chun Hyang University School of Medicine, Bucheon, Republic of Korea; ^4^Graduate School of International Agricultural Technology, Seoul National University, Pyeongchang, Republic of Korea

**Keywords:** streamlined culturomics, 16S rRNA gene amplicon sequencing, inflammatory bowel disease, ulcerative colitis, Crohn’s disease, gut microbiota

## Abstract

Inflammatory bowel disease (IBD) is characterized by disruptions in the gut microbiome. While most studies on gut dysbiosis in IBD rely on sequencing-based methods, we employed a streamlined culturomics approach to obtain a more comprehensive understanding of gut microbiota imbalance in patients with IBD that may not be captured by sequencing alone. A total of 367 bacteria were identified at the species level, including 211 species from ulcerative colitis patients, 164 species from Crohn’s disease (CD) patients, and 263 species from healthy individuals. Consistent with our 16S rRNA gene amplicon sequencing results, a significant decrease in microbial diversity and a severe imbalance, especially in CD patients, were also observed in the culture-based analysis. Our culturomics approach provided additional insights, highlighting dysbiosis in unique anaerobic and Gram-negative species in CD patients. Moreover, species-level findings for *Bifidobacterium* and *Enterobacterales* emphasized specific species expansions in IBD patients. Notably, *Mediterraneibacter gnavus*, *Thomasclavelia ramosa*, *Parabacteroides merdae*, and *Collinsella aerofaciens* are of particular clinical interest due to their correlation with inflammatory biomarkers. This comprehensive analysis underscores the value of integrating a culture-based approach with a genome-based approach to provide complementary insights and therapeutic targets in IBD.

## 1 Introduction

Inflammatory bowel disease (IBD), which encompasses Crohn’s disease (CD) and ulcerative colitis (UC), is a chronic and relapsing inflammatory disorder of the digestive tract characterized by disruptions in the gut microbiome (Glassner et al., 2020). UC primarily affects the colon and rectum, leading to mucosal inflammation and ulcers that can cause bloody stools. On the other hand, CD can inflame throughout the gastrointestinal tract, resulting in diarrhea and weight loss (Fakhoury et al., 2014).

The human gut microbiota is a diverse community of microorganisms that regulate human health through complex interactions with each other and their host (Thursby and Juge, 2017). Over the past few decades, next-generation sequencing (NGS)-based approaches have been instrumental in enhancing our understanding of the composition and functions of gut microbiota (Wang et al., 2015; Zhang et al., 2019; Gomaa, 2020). To date, numerous NGS analyses have been conducted to identify the microbial communities associated with gut dysbiosis in IBD (Franzosa et al., 2019; Lloyd-Price et al., 2019; Zuo et al., 2022; Wang et al., 2024). Most research has reported an increased abundance of *Pseudomonadota* (particularly *Enterobacteriaceae*) and decreased overall microbial diversity in IBD patients (Matsuoka and Kanai, 2015; Zhou et al., 2018). Additionally, patients with IBD have been found to have depleted populations of beneficial symbionts, such as *Bifidobacterium* spp., *Lactobacillus* spp., and *Faecalibacterium prausnitzii* (Caruso et al., 2020). Despite their effectiveness, however, these culture-independent approaches (e.g., Shotgun/16S rRNA gene amplicon sequencing) have several limitations. One major limitation is the sequencing depth bias, which has led to the underrepresentation of low-abundance bacteria (Lagier et al., 2018). In addition, around 70% of gut microbes remain uncultured, which means that a substantial proportion of the detected sequences have not yet been taxonomically identified (Almeida et al., 2021). Furthermore, NGS-based results typically do not reflect the viability of the detected microbes (Bellali et al., 2021), making it difficult to functionally characterize or experimentally verify them in a clinical or experimental setting. To address these limitations, there has been growing interest in culture-dependent gut microbiome approaches (Buttó et al., 2015; Lagier et al., 2018; Vrancken et al., 2019; Hitch et al., 2021).

Culturomics is a culturing strategy introduced in 2012 that involves high-throughput microbial cultivation using various culture conditions combined with identification techniques such as matrix-assisted laser desorption/ionization time-of-flight mass spectrometry (MALDI-TOF MS) and 16S rRNA gene sequencing (Lagier et al., 2012). Culturomics has demonstrated significant potential in expanding the repertoire of human gut bacterial species (Diakite et al., 2021), and it complements the insights provided by NGS-based approaches (Lagier et al., 2015; Diakite et al., 2021). Nevertheless, the labor-intensive nature of culturomics, particularly due to the use of multiple culture conditions, continues to limit its widespread application. Simplified culturomics conditions have been developed to make the process more feasible (Chang et al., 2019; Naud et al., 2020; Park et al., 2024), but culturomics has not yet been widely employed in disease contexts. Most studies have focused on healthy individuals (Diakite et al., 2019; Forster et al., 2019; Poyet et al., 2019; Mukhopadhya et al., 2022), and few have applied this method to investigate human gut dysbiotic states (Tidjani Alou et al., 2017; Dahal et al., 2023).

In this study, we aimed to comprehensively characterize the microbial signatures in both UC and CD patients by combining 16S rRNA gene amplicon sequencing with a streamlined culturomics protocol. The culturomics method employed here was based on limited culture conditions established in our previous research (Park et al., 2024).

## 2 Materials and methods

### 2.1 Subject information

This study included 41 patients under the medical follow-up by a gastroenterologist at Soon Chun Hyang University Bucheon Hospital between 2021 and 2022, consisting of 19 UC patients and 22 CD patients. The inclusion criteria were South Korean adults. They were restricted from taking antibiotics, probiotics, and fermented dairy products for at least 3 weeks before stool sampling. Pregnant women and those diagnosed with other diseases were excluded. Disease severity was assessed based on the Mayo score and Truelove–Witts severity score for UC patients (Truelove and Witts, 1955; Rubin et al., 2019) and the Crohn’s disease activity index for CD patients (Di Palma and Farraye, 2011). All participants provided their stool samples under their informed and signed consent. Additionally, the clinical database contained the blood test results obtained from the hospital for diagnostic purposes, along with patient information (Supplementary Data S1). This protocol was approved by the Institutional Review Board of Soon Chun Hyang University Bucheon Hospital (SCHBC 2021–01–028-002). To compare the differences in gut microbiota between each IBD group and healthy individuals, we applied the 16S rRNA gene amplicon sequencing results (*n* = 11) and culturomics findings (*n* = 8) from healthy subjects analyzed in our previous study to this study (Park et al., 2024). Healthy controls (HC) were defined as individuals with no medical history of gastrointestinal or metabolic diseases, recent antibiotic use, or consumption of fermented milk or probiotics.

### 2.2 Sample processing

To minimize changes in bacterial composition and viability in stool, we followed the Human Microbiome Project stool collection protocol (Wu et al., 2019) with some modifications (Supplementary Figure S1). Clinicians provided patients with sufficient information and education on how to store stools immediately after defecation at home. The samples were kept at a low temperature (< 4°C) and under anaerobic conditions with a GasPak EZ anaerobe container system (Becton Dickinson, MD, United States) until arrival at our laboratory. All samples were processed within 24 h of collection in an anaerobic chamber with a gas mixture of 5% CO_2_, 10% H_2_, and 85% N_2_ to eliminate oxygen. The shape and condition of stool samples were evaluated based on the Bristol stool scale and disease activity index, which included an assessment of stool consistency and visible blood. Stool consistency was scored as normal (0), loose stool (1), or diarrhea (2). Blood in the stool was scored as no visible blood (0), streaks of blood (1), apparent blood (2), or mostly blood (3) (Ogata et al., 2017).

A portion of the sample was placed in a collection tube for later quantification of stool inflammatory biomarkers (fecal calprotectin, FCP; fecal occult blood, FOB) using enzyme-linked immunoassay tests from CalproLab (Oslo, Norway) and Epitope Diagnostics Inc. (CA, United States), respectively. The remaining stool sample was homogenized and resuspended in saline at a concentration of 0.25 g/L. Some portions were stored at −80°C until DNA extraction and the rest were utilized for bacterial culturomics.

### 2.3 Bacterial isolation using a streamlined culturomics condition

We followed a streamlined culturomics protocol proposed in our previous study (Park et al., 2024). Fecal suspensions were immobilized into polysaccharides consisting of xanthan and gellan gums for a long-term incubation (Cinquin et al., 2004; Yeo et al., 2023; Park et al., 2024). The fecal gel beads were inoculated into preculture media at a final concentration of 5 g stool/L. The streamlined culturomics was conducted under aerobic and anaerobic conditions for 30 days at 37°C. These included BACT/ALERT FAN Plus Culture Bottles (BioMérieux, Marcy l’Etoile, France; FA PLUS Ref. 410851 for aerobic cultivation; FN PLUS Ref. 410852 for anaerobic cultivation) and modified Gifu Anaerobic Medium (mGAM; Nissui Pharmaceutical, Tokyo, Japan) (Park et al., 2024). All preincubation media were supplemented with 10% rumen fluid and 10% sheep blood. The cultured solutions were regularly taken (> six times for 30 days), serially diluted into saline, and inoculated on mGAM agar for bacterial isolation. After serial dilution, the fecal suspension without preincubation was also directly plated onto mGAM agar. Based on the colony morphology differences, colonies were randomly picked, inoculated into mGAM broth, and plated onto mGAM agar. Bacterial colonies were identified using MALDI-TOF MS on a MALDI Biotyper Sirius system (Bruker Daltonics, Bremen, Germany). If the signal of the bacterial spectrum was insufficient or did not match the MBT 8,468 MSPs library (score ≤ 1.69), we extracted genomic DNA using Chelex 100 resin (Bio-Rad Laboratories, CA, United States). The 16S rRNA gene was then sequenced using 27F (5′-AGAGTTTGATCCTGGCTCAG-3′) and 1492R (5′-GGTTACCTTGTTACGACTT-3′) primers using an ABI PRISM 3730XL DNA analyzer (Applied Biosystems, CA, USA) by SolGent (Daejeon, South Korea). Identified strains were preserved in 10% glycerol stock solution and stored at −80°C. The taxonomic lineage and names were updated based on the List of Prokaryotic names with Standing in Nomenclature database (LPSN).^1^ Species that showed the 16S rRNA gene sequence similarity of less than 98.65% with the closest neighbor were classified as potential novel species. A phylogenetic tree was constructed using PhyloT^2^ based on the 16S rRNA reference genes, which contained 360 species of this study. The tree was visualized using the iTOL tool^3^ (Letunic and Bork, 2024). We utilized the phenotypic characteristics of type strains registered in Bac*Dive*^4^ for oxygen tolerance and Gram characteristics. We also applied the bacterial oxygen tolerance database.^5^

Unlike the next-generation sequencing-based method that provides information on relative abundance based on ASV, the form of data obtained from culturomics is the number of isolated microorganisms under the given culture conditions. We followed the calculation methods for culturomics defined by Tidjani Alou et al. (2017) to compare isolated gut bacterial composition between groups. One of the key measures is the unique/total (U/T) ratio, which is the ratio of the number of unique species to the total species in each group. In this context, unique species were defined as bacteria isolated from only one subject in each group. Another critical measure is the relative frequency, the ratio of subjects in which a species is found to the total number of subjects in each group. For instance, if a species is isolated from all subjects, its relative frequency is 1.0; if it is found in only 10% of the subjects, its relative frequency is 0.1. Using these concepts, we calculated each species’ relative frequency differences (RFDs) by subtracting the HC group frequency from the UC or CD group frequency to identify species enriched or depleted in each IBD subtype. Additionally, we evaluated the differences in the number of isolates within specific bacterial taxa across groups.

### 2.4 16S rRNA amplicon sequencing and analysis

Genomic DNA was extracted from fecal samples using a ZymoBIOMICS DNA Miniprep Kit (Zymo Research, CA, United States) according to the manufacturer’s protocol. The sequence libraries were prepared according to Illumina’s instructions (Amplicon et al., 2013) and then sequenced by Macrogen (Seoul, South Korea) on an Illumina MiSeq system. We obtained 3,139,607 demultiplexed sequences with an average of 56,064 reads ranging from 35,562 to 76,011 reads per sample in the QIIME2 environment. The low-quality regions of the reads were denoised using the DADA2 pipeline (Callahan et al., 2016), and a rooted phylogenetic tree was generated. Taxonomic assignment was conducted using weighted naive classifiers trained on Silva 138.1 data specific to human stool samples (Kaehler et al., 2019). The QIIME2 files were further processed using the phyloseq package in R v4.2.2 (McMurdie and Holmes, 2013). The assigned features ranging from 25,024 to 52,733 were rarefied to 25,000-read sampling depth. The linear discriminant analysis (LDA) effect size (LEfSe) was used to select differential taxa in each IBD group compared with the HC group (Segata et al., 2011). The 16S rRNA gene amplicon sequences were deposited in the GenBank SRA database (PRJNA975689). Additionally, the SRA database for healthy controls (PRJNA975692 and PRJNA1094612) was imported and applied for comparison.

To quantify the bacterial cell number in stool samples, ZymoBIOMICS™ Spike-in Control I (Zymo Research, United States) containing equal cell numbers of two bacteria strains, *Imtechella halotolerans* and *Allobacillus halotolerans*, was added to each stool sample prior to DNA extraction. The protocol was followed according to the manufacturer’s instructions. The subsequent sequencing and analysis process was carried out in the same manner.

### 2.5 Statistical analysis

Statistical analyses were conducted in GraphPad Prism (v.10.2.2) and R software (v.4.3.2). Two-tailed Mann–Whitney test, two-sided Fisher’s exact test, and one-way ANOVA with Kruskal-Wallis test were used to analyze the clinical data and stool biomarkers. The two-sided Fisher’s exact test was applied to assess statistical significance in the U/T ratio and RFDs. Multiple Mann–Whitney test was used to analyze the number of cultured species in each phylum. One-way ANOVA with the Kruskal-Wallis test was used to analyze differences between groups in alpha diversity (Chao1 and InvSimpson indices), relative abundances at the phylum level, and bacterial cell counts in stool samples. The significance of the group distribution in beta diversity was determined by permutational multivariate analysis of variance (PERMANOVA) and permutational multivariate analysis of dispersion (PERMDISP). The correlation coefficients and statistical significance between the relative proportions (%) and inflammation biomarkers were calculated using Pearson correlation.

## 3 Results

### 3.1 Clinical characteristics

Our analysis included data from 41 IBD patients recruited for this study, along with data from 11 healthy individuals from our previous study (Park et al., 2024). Most of the 41 IBD patients analyzed were diagnosed with mild severity at the time of stool sample collection (58% with UC and 64% with CD) (Table 1). Most of the subjects in the UC group (95%, 18/19) had never been prescribed biologics, while a significant portion of the CD group (68%, 15/22) had a history of biologics treatment. While the ESR levels were similar between the two IBD groups, CRP levels were significantly higher in CD patients (4.6-fold, *p* = 0.03). Based on the morphological characteristics of the stool samples, we were able to classify samples as showing mushy or watery stool consistency or visible blood in the IBD groups. Notably, significantly higher levels of occult blood were quantified in the UC group compared to the HC group (*p* = 0.01). Moreover, the number of bacterial cells and FCP levels in the stool showed significant differences between the CD and HC groups, while the UC group exhibited intermediate values. Detailed clinical information is provided in Supplementary Data S1.

**Table 1:** Clinical characteristics.

	HC	UC	CD	UC vs. HC (*p*-value)	CD vs. HC (*p*-value)	CD vs. UC (*p*-value)
No. of subjects	11	19	22			
Age^a^	34.1 ± 2.0	49.6 ± 3.3	32.9 ± 2.0	**0.003**	0.55	**0.0002**
Female, *n* (%)^b^	5 (45%)	14 (74%)	5 (23%)	0.24	0.24	**0.002**
Severity, *n* (%)^b^						
Mild	**–**	11 (58%)	14 (64%)	–	–	0.76
Moderate	**–**	4 (21%)	7 (32%)	–	–	0.50
Severe	**–**	4 (21%)	1 (5%)	–	–	0.16
Biologic treatment, *n* (%)^b^						
Naïve	–	18 (95%)	7 (32%)	–	–	**< 0.0001**
Continuing	–	1 (5%)	14 (64%)	–	–	**< 0.0001**
Stopped	–	0 (0%)	1 (5%)	–	–	> 0.99
ESR (mm/h)^a^	–	24.6 ± 6.1	25.4 ± 6.5	–	–	0.85
CRP (mg/dL)^a^	–	0.3 ± 0.2	1.4 ± 0.6	–	–	**0.03**
Stool characteristics, bacterial quantification, and inflammatory biomarkers						
Bristol scale 6 or 7, *n* (%)^b^	0 (0%)	3 (16%)	6 (27%)	0.28	0.08	0.47
Stool DAI^c^	0.09 ± 0.09	0.95 ± 0.31	1.59 ± 0.38	0.05	**0.003**	0.22
Log (bacteria cells/g stool)^c^	11.08 ± 0.19	10.76 ± 0.10	10.6 ± 0.10	0.27	**0.03**	0.24
FCP (mg/kg stool)^c^	56.2 ± 13.3	509.1 ± 182.5	571.3 ± 141.1	**0.02**	**0.006**	0.60
FOB (μg/kg stool)^c^	0.9 ± 0.9	51.2 ± 17.9	19.8 ± 6.1	**0.01**	0.05	0.44

Statistically significant *p*-values (<0.05) are highlighted in bold.

Values are presented as number (%) or mean ± SEM. ESR, Erythrocyte sedimentation rate; CRP, C-reactive protein in blood; FCP, Fecal calprotectin; FOB, Fecal occult blood; Stool DAI, Stool disease activity index. HC data was retrieved from our previous study (Park et al., 2024).

a Two-tailed Mann–Whitney test.

b Two-sided Fisher’s exact test.

c One-way ANOVA using Kruskal–Wallis test.

### 3.2 Culture-independent analysis of gut dysbiosis in patients with IBD

**Figure 1:**
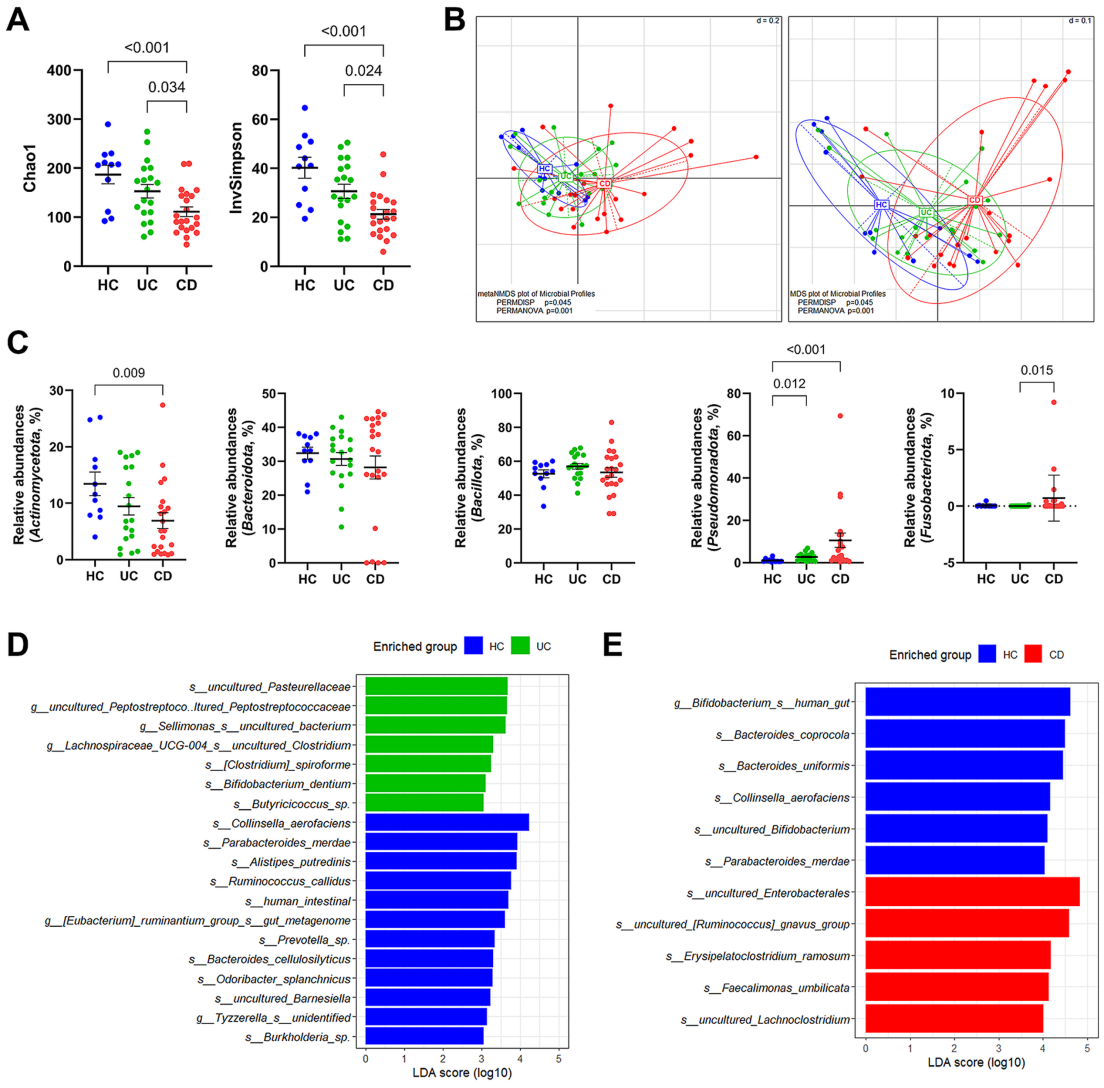
Taxonomic profile of gut microbiota in patients with IBD via 16S rRNA gene amplicon sequencing. **(A)** Alpha diversity estimated Chao1 and InvSimpson. **(B)** NMDS and MDS plots of samples. PERMDISP and PERMANOVA were used to statistically evaluate significant differences. **(C)** Group comparison of the relative abundances at the phylum level. **(D)** LEfSe analysis between the UC and HC groups. The LEfSe analysis was computed with a *p* value cutoff of 0.05 for the Kruskal–Wallis (KW) test and an LDA score cutoff of 3. **(E)** LEfSe analysis between the CD and HC groups. Only taxa meeting a LDA threshold value of >4.0 and having a *p*-value of <0.05 are shown. **(A,C)** *p*-values were calculated using one-way ANOVA with Kruskal-Wallis test, and values lower than 0.05 were indicated.

We compared the microbiota composition between the HC and IBD groups via 16S rRNA gene amplicon sequencing. In the alpha diversity analysis estimated Chao1 and InvSimpson indices, IBD patients exhibited significantly low microbial diversities compared to the HC group (Figure 1A). This tendency was also observed in the beta diversity based on the phylogenetic distance among communities, where the three groups showed significant group distribution (PERMANOVA *p* = 0.001 and PERMDISP *p* = 0.045) (Figure 1B). Notably, the CD group showed a distinct cluster from the HC group. In contrast, the UC group was distributed widely, overlapping with the HC and CD groups. At the phylum level, we observed significantly higher relative abundances of *Pseudomonadota* in both IBD subtypes and decreased *Actinomycetota* populations in CD patients (Figure 1C). Additionally, *Fusobacteriota* was detected in 40.9% (9/22) of CD patients. LEfSe analysis was conducted to compare the relative contributions of different taxa using an adjusted *p-*value cutoff of 0.05 for the Kruskal–Wallis test and an LDA score cutoff of 3.0. We identified 19 different taxa at the species level between the UC and HC groups, of which seven were differentially enriched in the UC group (Figure 1D; Supplementary Table S1). In contrast, a total of 55 differential taxa were identified in pairwise comparisons between the CD and HC groups (Figure 1E; Supplementary Table 2). Among them, 10 species were enriched in the CD group. The entire taxa list, detailed LDA score, *p-*values, and adjusted *p-*values are described in Supplementary Tables S1, S2.

### 3.3 Culture-dependent analysis of gut dysbiosis in patients with IBD

**Figure 2:**
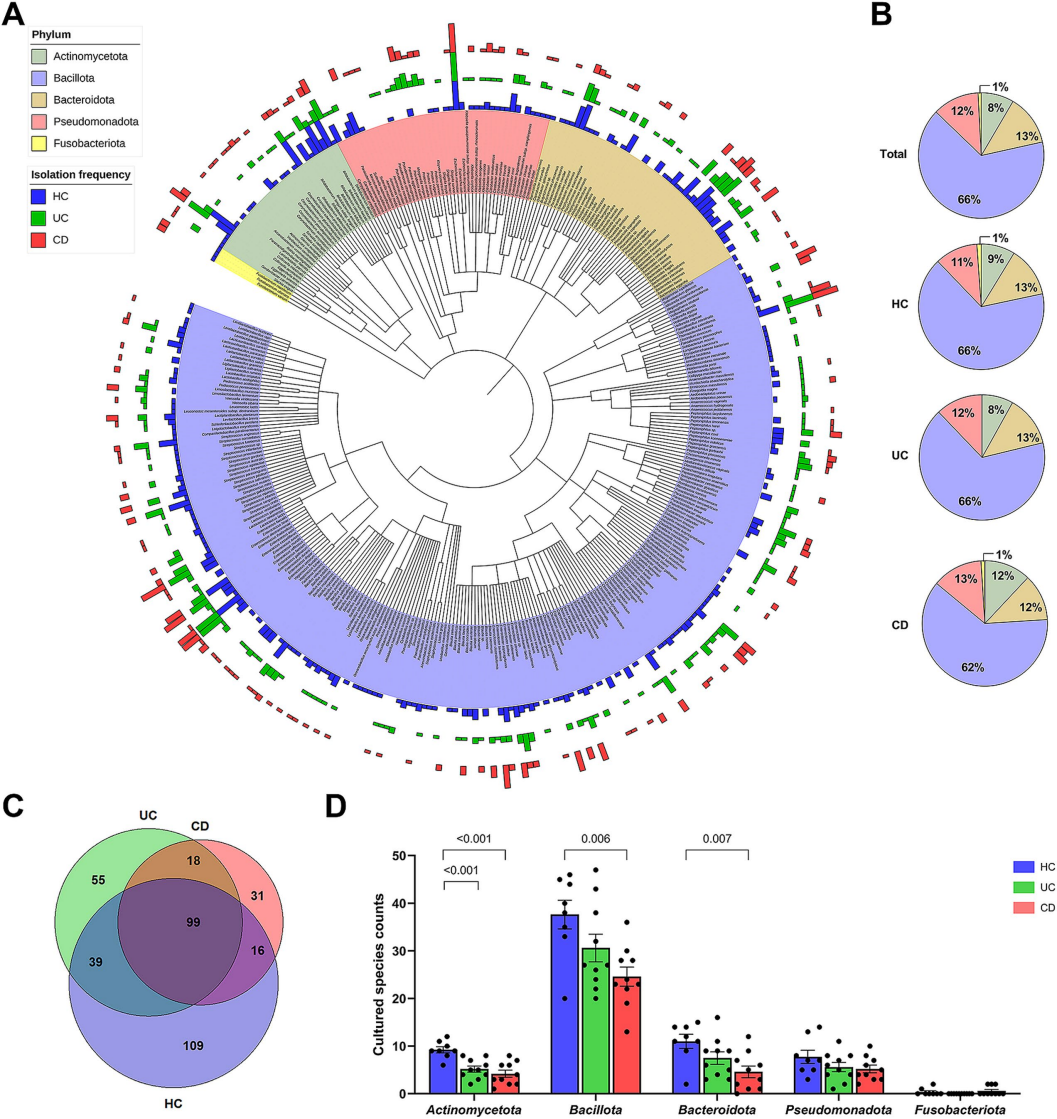
Cultured gut bacterial characteristics between groups. **(A)** A phylogenetic tree based on the available 16S rRNA gene sequences of 360 bacterial species from a total of 367 cultured bacterial species. Bars in the outer circles indicate the isolation frequency of each species within each group, with the order being CD, UC, and HC from outer to inner circles. Each phylum is represented by a distinct color. **(B)** Composition of cultured bacterial species at the phylum level, showing relative proportions of each phylum within each group. **(C)** Venn diagram showing the number of shared and exclusively isolated species across groups. **(D)** Comparison of cultured species counts at the phylum level. Statistical significance was calculated with multiple Mann–Whitney tests, with values lower than 0.05 indicated.

Among the collected stool samples from IBD patients, 53.8% (28/52) of samples were included in the culturomics analysis. Ten samples from each of the UC and CD groups were used for cultivation (Supplementary Table S3). Additionally, culturomics data of eight healthy individuals from our previous study were included (Park et al., 2024). The culturomics cohort was comparable to the entire cohort in terms of clinical characteristics, inflammatory biomarkers, and 16S rRNA gene amplicon sequencing results (Supplementary Table S3; Figure 2). Our cultured strain libraries included a total of 14,095 isolates, consisting of 6,134 isolates from the UC patients and 7,961 isolates from the CD patients. The gut bacterial library of UC patients was classified into 211 species, corresponding to four phyla, 11 classes, 15 orders, 40 families, and 91 genera (Supplementary Data S2). The gut bacterial library of CD patients was classified into 164 species, corresponding to five phyla, 12 classes, 18 orders, 35 families, and 72 genera (Supplementary Data S3). From IBD patients, three species, including *Selenobaculum gibii* gen. Nov. sp. nov. (Yeo et al., 2023), were classified as potential novel species (Supplementary Table S4).

The combined dataset of 258 species from the IBD groups and 263 species from the HC group yielded a total of 367 species (Figure 2A). The number of subjects from whom each species was isolated, i.e., the isolation frequency, showed diverse patterns at the species level (Outer circle in Figure 2A). The species isolated from over 70% of subjects in each group were *Enterococcus faecium*, *Clostridium innocuum*, and *Escherichia coli*. The human gut bacterial library was comprised of *Bacillota* (65.7%), *Bacteroidota* (13.1%), *Pseudomonadota* (12.0%), *Actinomycetota* (8.4%), and *Fusobacteriota* (0.8%) (Figure 2B). The overall phylum composition in the UC group was similar to that of the HC group, except for the absence of *Fusobacteriota*. In contrast, the CD group showed the relative composition of phylum *Bacillota* (62.2%), *Bacteroidota* (11.6%), *Pseudomonadota* (12.8%), *Actinomycetota* (12.2%), and *Fusobacteriota* (1.2%). The number of exclusively isolated species was highest in the HC group (109 species), followed by the UC (55 species) and CD groups (31 species) (Figure 2C). The species diversity of *Actinomycetota* was significantly lower in both IBD groups compared to the HC group (Figure 2D). Similarly, the species diversities of *Bacillota* and *Bacteroidota* were significantly lower in the CD group. Moreover, our U/T ratio comparison revealed that the isolation of unique bacterial species was lower in the IBD groups compared to the HC group, with the CD group, in particular, showing significantly lower unique anaerobes (−18.9%, *p* = 0.008) and Gram-negative populations (−18.9%, *p* = 0.04) (Table 2).

**Table 2:** Comparison of Unique/Total (U/T) ratios of cultured gut bacterial diversity between the healthy and IBD groups.

	HC (*n* = 8)		UC (*n* = 10)		CD (*n* = 10)		△Diversity^a^ (*P-*value^b^)			
							UC vs. HC		CD vs. HC	
U/T ratio based on the presence of oxygen tolerance										
All species	153/263	(58%)	113/211	(54%)	80/164	(49%)	−4.6%	(0.35)	−9.4%	(0.07)
Anaerobic species	76/137	(55%)	61/116	(53%)	30/82	(37%)	−2.9%	(0.70)	**−18.9%**	**(0.008)**
Aerotolerant species	77/126	(61%)	52/95	(55%)	50/82	(61%)	−6.4%	(0.41)	−0.1%	(>0.99)
U/T ratio based on the Gram characteristics										
Positive	103/179	(58%)	79/147	(54%)	61/116	(53%)	−3.8%	(0.50)	−5.0%	(0.47)
Negative	47/81	(58%)	34/63	(54%)	18/46	(39%)	−4.1%	(0.74)	**−18.9%**	**(0.04)**
Variable	3/3	(100%)	0/1	(0%)	1/2	(50%)	−100.0%	(0.25)	−50.0%	(0.40)

Statistically significant △Diversity values and *p*-values (<0.05) are highlighted in bold.

a △Diversity means the values obtained by subtracting the diversity of the healthy controls from the disease group.

b Two-sided Fisher’s exact test.

### 3.4 Culturomics-based differential bacterial species in patients with IBD

Next, we identified bacterial species showing differences between the HC and each IBD group by calculating RFDs based on the isolation frequency of cultured species (Table 3). There were notable differences in the microbial signature patterns between the UC and CD groups. In the pairwise analysis between the UC and HC groups, 12 species were identified as statistically significant, with *Bifidobacterium dentium*, *Enterococcus gallinarum*, *Flavonifactor plautii*, and *Peptoniphillus gorbachii* being enriched in the UC group. In contrast, the pairwise analysis between the CD and HC groups revealed differences in 16 species, with *Mediterraneibacter gnavus*, *Thomasclavelia ramosa*, and *Morganella morganii* being enriched in the CD group. Consistent with our findings, the previous LEfSe analysis also identified *B. dentium*, *M. gnavus*, and *T. ramosa* as taxa, showing differential abundances in each IBD group, further supporting their potential role in IBD pathogenesis (Figures 1D,E; Supplementary Tables S1, S2). The culturomics analysis revealed 13 species with significantly lower RFDs in the CD group compared to the HC group. Among these, *B. bifidum*, *Collinsella aerofaciens*, *Phocaeicola coprocola*, and *Parabacteroides merdae* were also identified as depleted taxa in the CD group compared to the HC group through LEfSe analysis (Figure 1E; Supplementary Table S2). Intriguingly, *C. aerofaciens* was consistently depleted in both UC and CD patients, as revealed by both culturomics and LEfSe analysis (Figures 1D,E).

**Table 3:** Relative frequency differences (RFDs) between the IBD groups and the HC group.

Phylum/Genus	Species	RFDs (*P*-value^a^)			
		UC vs. HC		CD vs. HC	
*Actinomycetota*					
*Bifidobacterium*	*Bifidobacterium catenulatum*	**−0.8**	**(0.001)**	**−0.8**	**(0.001)**
	*Bifidobacterium adolescentis*	**−0.6**	**(0.01)**	**−0.8**	**(0.001)**
	*Bifidobacterium pseudocatenulatum*	**−0.6**	**(0.01)**	**−0.7**	**(0.004)**
	*Bifidobacterium longum* subsp. *suillum*	**−0.5**	**(0.04)**	**−0.5**	**(0.04)**
	*Bifidobacterium bifidum*	−0.1	(0.66)	**−0.5**	**(0.04)** ^**#**^
	*Bifidobacterium dentium*	**+0.6**	**(0.02)** ^**#**^	**+**0.4	(0.15)
*Collinsella*	*Collinsella aerofaciens*	**−0.7**	**(0.004)** ^**#**^	**−0.7**	**(0.004)** ^**#**^
*Bacillota*					
*Bacillus*	*Bacillus cereus*	−0.2	(0.64)	**−0.5**	**(0.04)**
*Enterococcus*	*Enterococcus gallinarum*	**+0.7**	**(0.01)**	**+**0.5	(0.15)
*Latilactobacillus*	*Latilactobacillus sakei*	−0.4	(0.12)	**−0.5**	**(0.02)**
*Mediterraneibacter*	*Mediterraneibacter gnavus*	**+**0.1	(>0.99)	**+0.6**	**(0.02)** ^**#**^
*Flavonifractor*	*Flavonifractor plautii*	**+0.6**	**(0.02)**	**+**0.4	(0.15)
*Peptoniphilus*	*Peptoniphilus gorbachii*	**+0.5**	**(0.04)**	**+**0.1	(>0.99)
*Thomasclavelia*	*Thomasclavelia ramosa*	**+**0.2	(0.59)	**+0.6**	**(0.02)** ^**#**^
*Bacteroidota*					
*Phocaeicola*	*Phocaeicola coprocola*	−0.4	(0.12)	**−0.5**	**(0.02)** ^**#**^
*Alistipes*	*Alistipes shahii*	−0.3	(0.34)	**−0.6**	**(0.01)**
*Parabacteroides*	*Parabacteroides merdae*	−0.4	(0.15)	**−0.7**	**(0.02)** ^**#**^
*Pseudomonadota*					
*Mesosutterella*	*Mesosutterella multiformis*	**−0.5**	**(0.02)**	**−0.5**	**(0.02)**
*Sutterella*	*Sutterella wadsworthensis*	**−0.8**	**(0.002)**	−0.4	(0.19)
*Campylobacter*	*Campylobacter ureolyticus*	**−0.5**	**(0.04)**	**−0.6**	**(0.01)**
*Morganella*	*Morganella morganii*	**+**0.4	(0.09)	**+0.5**	**(0.04)**

Statistically significant RFD values and *p*-values (<0.05) are highlighted in bold.

a Two-sided Fisher’s exact test.

^#^Differential taxa selected in LEfSe analysis (adjusted *p*-value < 0.05 and LDA score > 3).

### 3.5 Culturomics insights into species-level profiling of Bifidobacterium and Enterobacterales in Crohn’s disease

In this study, culturomics complemented 16S rRNA gene amplicon sequencing by providing insights into culturable species diversity, while sequencing offered information on relative abundances. Results showed a significant reduction in the relative abundances and culturable species diversity of *Actinomycetota* in the CD group compared to the HC group (Figures 1C, 2D). While *Pseudomonadota* exhibited an overall increase in relative abundances in the CD group, their cultured species diversity did not significantly differ from that of the HC group, suggesting an expansion of specific *Pseudomonadota* species (Figures 1C, 2D). Furthermore, the LEfSe analysis revealed that *Bifidobacterium* and *Enterobacterales* were the taxa that best differentiated between the HC and CD groups, belonging to the phyla *Actinomycetota* and *Pseudomonadota*, respectively (Figure 1E; Supplementary Table S2).

**Figure 3:**
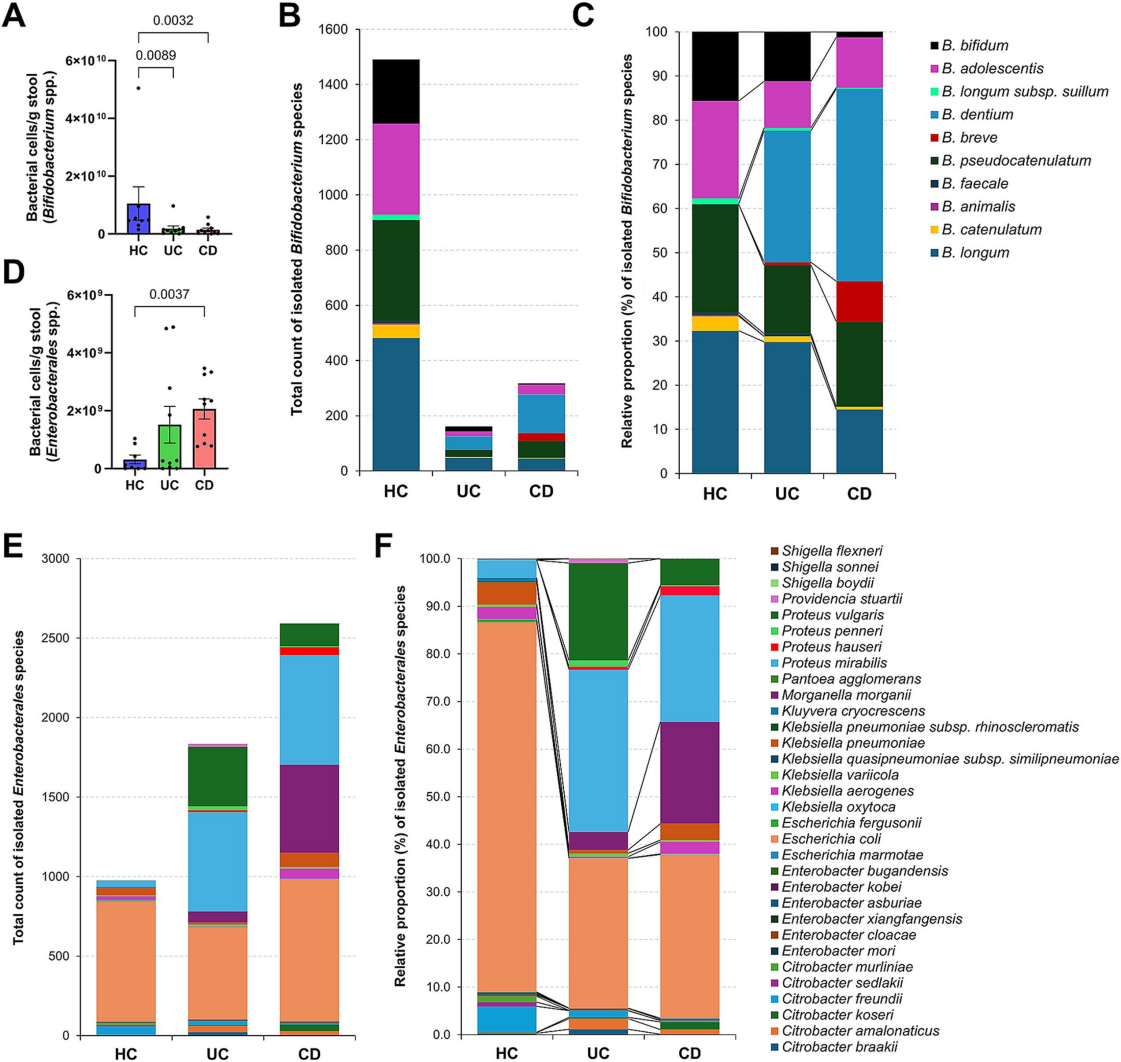
Culturomics-based species signatures of *Bifidobacterium* and *Enterobacterales* in Crohn’s disease. **(A)** The number of *Bifidobacterium* cells quantified using ZymoBIOMICS™ Spike-in Control I in initial stool samples. **(B)** Total number of *Bifidobacterium* species isolates. **(C)** Relative proportion (%) of isolated *Bifidobacterium* species within groups. **(D)** The number of *Enterobacterales* cells quantified using ZymoBIOMICS™ Spike-in Control I in initial stool samples. **(E)** Total number of *Enterobacterales* species isolates. **(F)** Relative proportion (%) of isolated *Enterobacterales* species within groups. **(A,D)** *p*-values were calculated using one-way ANOVA with Kruskal-Wallis test, with values lower than 0.05 indicated.

Given the limitations of species-level classification in 16S rRNA gene amplicon analysis and the high sequence similarity within the order *Enterobacterales* (Gupta et al., 2019; Gupta et al., 2019), it is challenging to obtain additional information on the species-level diversity of *Bifidobacterium* and *Enterobacterales*. When estimating bacterial cell numbers in the spiked initial cultured stool samples, the CD group exhibited significantly lower *Bifidobacterium* levels compared to the HC group (average 1.4 × 10^9^ cells/g stool vs. 1.0 × 10^10^ cells/g stool) (Figure 3A). This finding was corroborated by the culture-based analysis, where 1,491 isolates (nine species) were obtained from healthy individuals (*n* = 8), whereas only 317 isolates (eight species) were obtained from CD patients (*n* = 10) (Figure 3B). When examined as relative proportions within each group, distinct patterns were observed for each species. Specifically, *B. dentium* and *B. breve* exhibited a greater increase in proportion in the CD group compared to the HC group (Figure 3C). Additionally, the remaining *Bifidobacterium* species represented lower proportions in the CD group compared to the HC group, particularly for *B. bifidum* (*p* = 0.0002) and *B. longum* (*p* = 0.007).

The spiked initial fecal samples from CD patients exhibited a higher number of *Enterobacterales* cells compared to healthy individuals (average 2.1 × 10^9^ cells/g stool vs. 3.1 × 10^8^ cells/g stool) (Figure 3D). The culture-based analysis yielded a similar trend, with CD patients showing approximately 2.7 times more isolates (2,593 isolates; 17 species) than the HC group (976 isolates; 23 species) (Figure 3E). For *E. coli*, which was isolated from all subjects (Figure 2A), the isolation count was similar between the groups (Figure 3E). However, its relative proportion was significantly higher in the HC group (78% of *Enterobacterales*) (Figure 3F). In contrast, *Proteus vulgaris* (*p* = 0.03), *Proteus mirabilis* (*p* < 0.0001), and *M. morganii* (*p* < 0.0001) showed significant increases in both isolation counts and relative proportions in the CD group, suggesting a potential role in the pathogenesis of CD (Figure 3F).

### 3.6 Bacterial species associated with inflammation biomarkers in IBD

**Figure 4:**
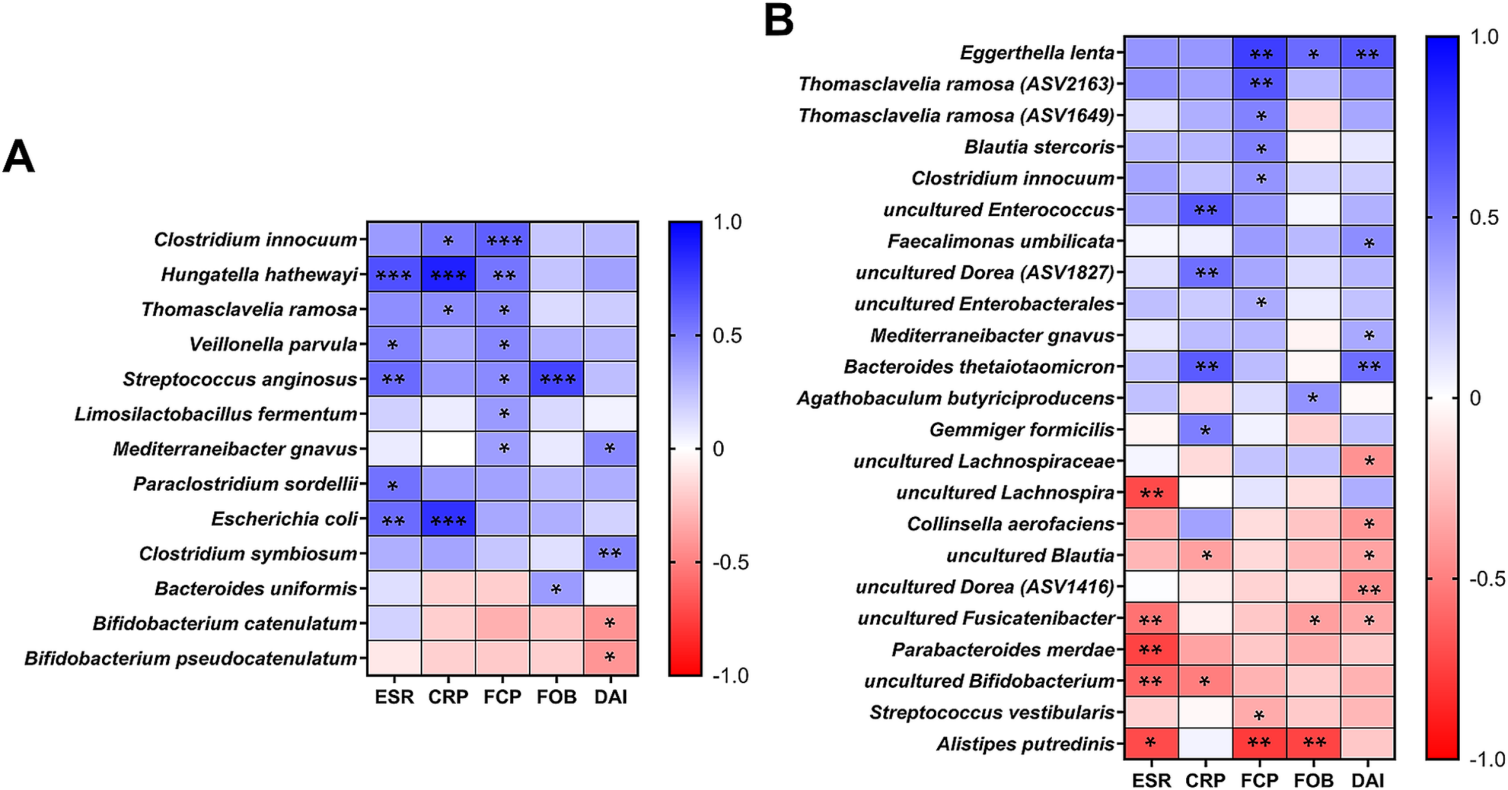
Correlations between bacterial species and inflammation biomarkers. **(A)** Pearson correlations between culturomics-derived relative proportions (%) and inflammatory biomarkers (ESR, CRP, FCP, FOB, and stool DAI). **(B)** Pearson correlations between 16S rRNA gene amplicon sequencing-derived relative abundances (%) and inflammatory biomarkers. **(A,B)** For both analyses, a prevalence cutoff of species present in >30% of total samples was applied. Statistical significance is indicated as follows: *p < 0.05; **p < 0.01; ***p < 0.001.

We further investigated bacterial species correlated with both blood (ESR, CRP) and fecal (FCP, FOB, Stool DAI) inflammatory biomarkers, applying a prevalence cutoff for species present in more than 30% of total samples (Figure 4). In our culturomics analysis, significant correlations were identified with 13 bacterial species (Figure 4A). Among these, *B. pseudocatenulatum* and *B. catenulatum* had significant negative correlations with stool DAI scores (*p* = 0.028 and 0.024, respectively). From the 16S rRNA gene amplicon sequencing results (Figure 4B), unidentified *Bifidobacterium* populations showed significant negative correlations with the blood inflammation biomarkers (*p* = 0.002 for ESR and 0.012 for CRP). Additionally, *P. merdae* and *C. aerofaciens*, which showed differences in relative frequency across groups in our culturomics results (Table 3), exhibited negative correlations with ESR (*p* = 0.003) and stool DAI scores (*p* = 0.03), respectively, when analyzed using the relative abundances (Figure 4B). Among taxa showing positive correlations with the biomarkers, *C. innocuum*, *T. ramosa*, and *M. gnavus* were commonly selected from both approaches (Figure 4). When considering their enrichments in CD patients (Figure 1E; Table 3; Supplementary Table S2), the observed correlations suggest a strong association with increased fecal inflammation as indicated by higher stool DAI scores and FCP values in CD patients (Table 1).

## 4 Discussion

Multiple studies have consistently demonstrated perturbations in the gut microbiota of patients with IBD (Franzosa et al., 2019; Lloyd-Price et al., 2019; Zuo et al., 2022; Wang et al., 2024). While most of the research has been genome-based, recent advances in high-throughput culture-based methods have shown valuable potential in addressing the lack of strain resources needed for further study (Forster et al., 2019; Poyet et al., 2019). These culture-based approaches offer additional findings or insights that complement the information provided by genome-based methods alone (Tidjani Alou et al., 2017; Diakite et al., 2019). In this context, this study was conducted to gain a better understanding of gut dysbiosis in UC and CD patients by implementing a culturomics approach. To ensure consistency and comparability with our previously obtained culturomics data of healthy individuals, we employed the same procedures that avoided inducing the dominance of specific microbial communities (Park et al., 2024). Furthermore, to ensure robust analysis of culturomic data, which relies on the number of isolated strains and species, we focused on maximizing the number of isolates per sample. As a result, our cultured isolate collection exhibited a broad phylogenetic diversity, providing extended taxonomic information that complemented the 16S rRNA gene amplicon sequencing results (Figure 2A).

Our 16S rRNA gene sequencing analysis demonstrated reduced microbial diversity in both IBD subtypes compared to the HC group (Figure 1A), consistent with findings from previous studies (Franzosa et al., 2019). The distinct clustering of the CD gut microbiota in beta diversity indicates a unique dysbiotic profile (Figure 1B), further characterized by an increased abundance of *Pseudomonadota* and a decreased abundance of *Actinomycetota* (Figure 1C). A similar tendency was observed in our culturomics analysis. The lower total number of isolated species and the reduced number of exclusively cultured species in IBD patients, particularly in CD patients, support a decrease in microbial diversity (Figure 2). The U/T ratio analysis provided further insights, showing the reduced diversity of unique anaerobes in CD patients, which indicated dysanaerobiosis (Table 2). Dysanaerobiosis refers to a disruption of the typical anaerobic environment within the gut. According to Rigottier-Gois (2013), increased oxygen levels can disrupt anaerobiosis, creating a selective advantage for facultative anaerobes or aerobes, allowing them to overgrow, while obligate anaerobes, which are sensitive to oxygen, are disadvantaged. Our findings support the “oxygen hypothesis,” which posits that increased luminal oxygen levels are a key contributor to IBD dysbiosis (Rigottier-Gois, 2013). This mucosal oxygen imbalance induces an abundance of facultative anaerobes relative to obligate anaerobes, and the overgrowth of *Enterobacteriaceae* is a representative example (Zeng et al., 2017). Our result aligns with Shahir et al.’s work on the importance of the aerotolerant species profile in CD patients (Shahir et al., 2020). Therefore, the depleted anaerobic species (e.g., *Bifidobacterium* spp.) in CD patients may serve as potential candidates for restoring intestinal dysanaerobiosis (Table 3; Figure 3C). Meanwhile, IBD patients have been frequently reported to exhibit a higher proportion of Gram-negative species and a lower proportion of Gram-positive species (Loh and Blaut, 2012). While the diversity of species within these Gram categories has been less explored, our results demonstrated a notable reduction in the diversity of unique Gram-negative species in CD patients (Table 2).

Numerous studies have aimed to identify specific microbial alterations in IBD, highlighting the importance of differential bacterial species (Schirmer et al., 2019). Our integrated analysis identified *M. gnavus* and *T. ramosa* as enriched in CD patients (Table 3). These species consistently demonstrated an association with inflammation biomarkers, particularly with fecal calprotectin (Figure 4). The expansion of *M. gnavus* has been reported in CD patients, but a causal relationship remains to be elucidated (Crost et al., 2023). A recent study revealed that one clade of *M. gnavus* harbors genes adapted to the IBD gut ecology (Hall et al., 2017). Similarly, another study demonstrated that *T. ramosa* isolated from patients with IBD exhibited direct genotoxicity (Cao et al., 2022). These findings highlight not only the need for further investigation at the strain level but also the need to secure strain resources isolated from disease-specific patient populations. Furthermore, our culturomics analysis has revealed the enrichment of additional species in CD patients: *M. morganii*, *P. mirabilis*, and *P. vulgaris* (Table 3; Figure 3F). The relationship between genotoxin molecule (indolimines)-producing *M. morganii* and colonic tumor development has been confirmed in an experimental mouse model (Cao et al., 2022). *P. mirabilis* may play a key role in the pathogenesis of CD, contributing to the induction of inflammation in animal colitis models (Zhang et al., 2021).

Our NGS and culturomics analyses revealed an enrichment of *B. dentium* in UC patients. Although *B. dentium* is generally considered beneficial due to its role in fermenting dietary carbohydrates and producing short-chain fatty acids, its elevated abundance in UC patients raises questions about its potential role in the disease (Engevik et al., 2021; Bosselaar et al., 2024). We also observed high levels of isolation frequency in *E. gallinarum* in UC patients. Previous studies have shown the potential of *Enterococcus* species in antibiotic resistance and rapid adaptation to the environment in IBD (Růžičková et al., 2020; Yang et al., 2022). Our culturomics findings in a high prevalence of *F. plautii* in UC patients are consistent with previous NGS-based studies (Pisani et al., 2022). Given that flavonoids are known to alleviate the severity of various inflammatory diseases, the high abundance of flavonoid-degrading *F. plautii* in IBD patients has been associated with increased intestinal inflammation (Gupta et al., 2019; Gupta et al., 2019). Furthermore, *P. gorbachii* was frequently isolated from UC patients in our study. Although prior research suggested that *P. gorbachii* may improve gut homeostasis and immune regulation by alleviating collagen-induced arthritis in mice (Kim et al., 2024), its clinical significance in IBD remains elucidated.

Among the depleted bacterial species in IBD patients, *C. aerofaciens* was notable due to its significant decrease in the UC and CD groups (Figures 1D,E; Table 3) and its negative correlation with stool DAI scores (Figure 4B). While some reports demonstrated a decrease *C. aerofaciens* population in the UC patients, the clinical significance of this finding remains controversial and requires further investigation (Joossens et al., 2011; Quagliariello et al., 2020; Kwon et al., 2023). Likewise, although the scientific understanding of *P. merdae*’s role in the human gut is limited, its ability to degrade intestinal branched-chain amino acids (BCAAs) (Qiao et al., 2022), coupled with the known role of BCAAs in exacerbating colitis symptoms via the mTOR/p70S6K pathway in mice (Huang et al., 2024), suggests that *P. merdae* may be a promising therapeutic target for IBD. Additionally, the low isolation frequency of *P. coprocola* in CD patients, which aligns with a previous report showing its higher abundance in healthy individuals (Nomura et al., 2021), suggests its potential as a candidate for further investigation regarding its clinical relevance with IBD. A depletion of *Bifidobacterium* species, except for *B. dentium* and *B. breve*, was common in both IBD subtypes (Figure 3C). Thus, further in-depth analysis of *Bifidobacterium* species at the strain level, taking into account host- and strain-specificity, is needed using strains obtained through cultivation (Bosselaar et al., 2024).

Although culturomics has significantly improved the efficiency of high-throughput cultivation, it remains a labor-intensive and time-consuming process, which limits its broader application (Lagier et al., 2018). Therefore, more efficient strategies and technologies are needed to accommodate large sample sizes. The expanded sample sizes would facilitate the acquisition of culture-based knowledge for studies such as longitudinal investigations to capture the microbiome dynamics of disease progression (Halfvarson et al., 2017), the responses of gut microbiota to clinical interventions, and analyses of mucosa-adherent bacteria from multiple gastrointestinal regions (Shahir et al., 2020; Mukhopadhya et al., 2022; Pu et al., 2022). Our study has a limitation related to cohort composition. While the gender and age distribution of the clinical subjects appears to align with the 2019 prevalence survey results for Korean IBD patients (Korean Association for the Study of Intestinal Diseases, 2020), potential biases may arise from the use of biologics, particularly in the CD group. We conducted a subgroup analysis based on biologics treatment but did not find significant differences in gut microbiota composition (Supplementary Figure S3). In addition, there were no considerable correlations between biologics and other clinical variables (Supplementary Figure S4). Nevertheless, various factors, such as the type of biological agent and the duration of treatment, can influence the gut microbiota (Estevinho et al., 2020). Another limitation of our study lies in the simplified cultivation condition we used. These conditions were designed to broadly capture diverse bacterial species; however, this approach may have excluded microorganisms with specific growth requirements, such as microaerophilic or slow-growing bacteria, which are known to be associated with IBD pathogenesis (Eckburg and Relman, 2007; Hansen et al., 2013). Future studies incorporating tailored culturomics protocols to target such challenging organisms would provide a more comprehensive understanding of gut dysbiosis in IBD.

In conclusion, our study highlights the complementary strengths of culturomics to 16S rRNA gene amplicon sequencing in gut microbiome research, providing an expanded perspective on gut dysbiosis in patients with IBD. Notably, dysbiosis in CD patients was characterized by reduced microbial diversity and unique obligate anaerobes. We identified enriched and depleted species in each IBD subtype based on the cultured species collection. The established large-scale collection will also serve as a valuable resource for understanding human gut bacterial diversity and strain-specific characteristics. Strain-level insights will be an important source for advancing targeted microbiome-based therapeutics in IBD. In addition, establishing an integrated experimental platform with a cultivation-based approach capable of comprehensively analyzing the host-specificity, microbial or microbial-to-host interactions, and metabolites of each microbe in the gut microbiota should be considered.

## Data Availability

The 16S rRNA gene amplicon sequences were deposited in the GenBank SRA database (PRJNA975689). The genome sequences of potential novel species were deposited in the GenBank under BioProject no. PRJNA1077756 and the specific accession numbers were provided in Supplementary Table S4. The 16S rRNA gene sequences of identified isolates were deposited in the GenBank and the specific accession numbers were provided in Supplementary Data S2 and S3. The SRA database for healthy controls (PRJNA975692 and PRJNA1094612) was imported and applied for comparison.

## Footnotes

^1^ https://lpsn.dsmz.de/

^2^ https://phylot.biobyte.de/

^3^ https://itol.embl.de/

^4^ https://bacdive.dsmz.de/

^5^ https://www.mediterranee-infection.com/acces-ressources/base-de-donnees/list-of-prokaryotes-according-to-their-aerotolerant-or-obligate-anaerobic-metabolism/
