# Supplementary material for: Culture-based characterization of gut microbiota in inflammatory bowel disease
Source: Front Microbiol. 2025 Feb 20;16:1538620. doi: 10.3389/fmicb.2025.1538620 (PMC11884817; doi:10.3389/fmicb.2025.1538620)
Supplement: Supplementary file 1 [file Data_Sheet_1.ZIP › Supplementary_Materials/Supplementary_Figures.pdf]

Supplementary Figures

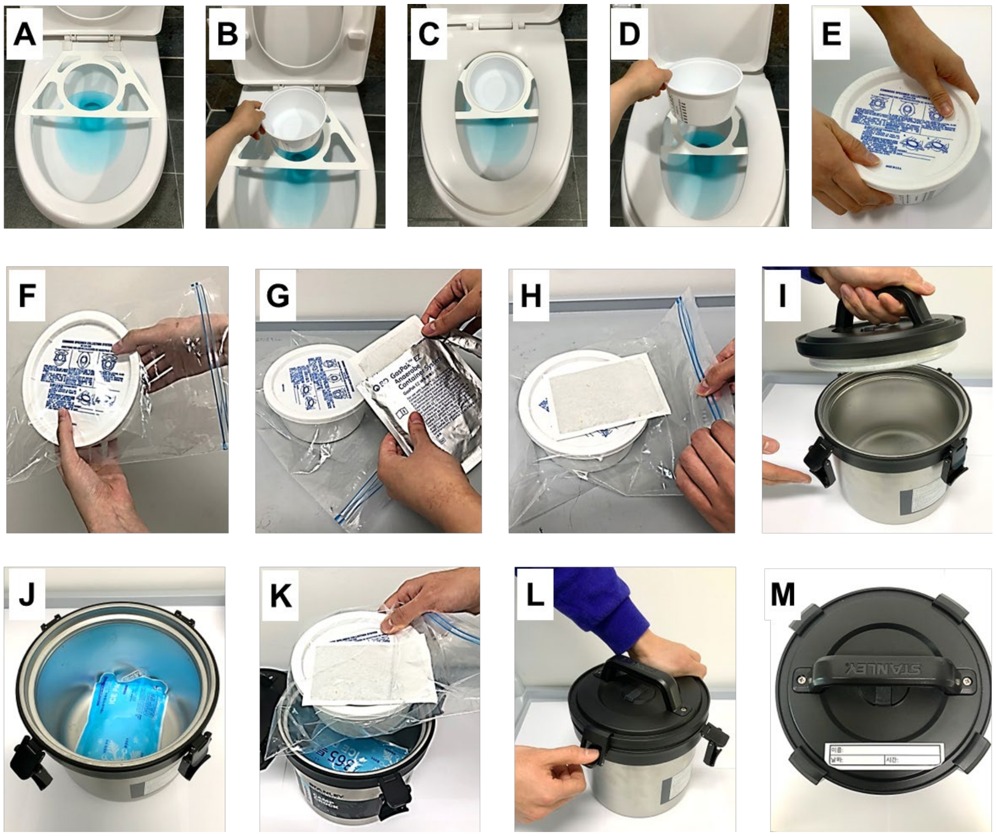

Supplementary Figure 1. Stool sample collection procedure. A to E: Setup procedure of the stool collection container. **F to H:** Creating anaerobic conditions using a GasPak EZ anaerobic pouch. **I to K:** Placing an ice pack to maintain low temperature until arrival (< 24 h) at the laboratory. **L to M:** Sealing the sample in a vacuum container.

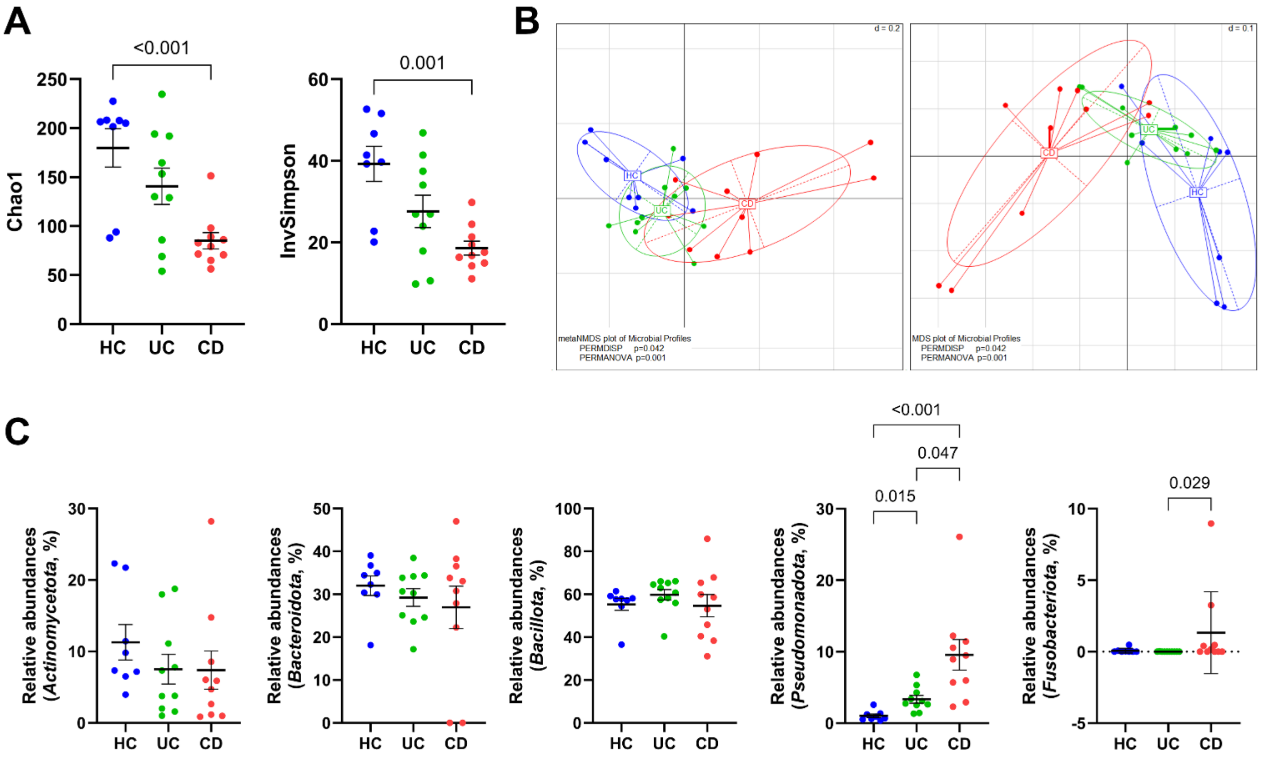

Supplementary Figure 2. Gut microbiota composition of the culturomics cohorts via 16S rRNA gene amplicon sequencing (*n* = 28). A alpha diversity was estimated by Chao1 and InvSimpson indices. **B** Beta diversity was estimated using NMDS and MDS plots with PERMDISP ($p = 0.042$) and PERMANOVA ($p = 0.001$). **C** Relative abundances at the phylum level. **A, C** One-way ANOVA with Kruskal-Wallis test was used to analyze differences between groups and P values lower than 0.05 were indicated.

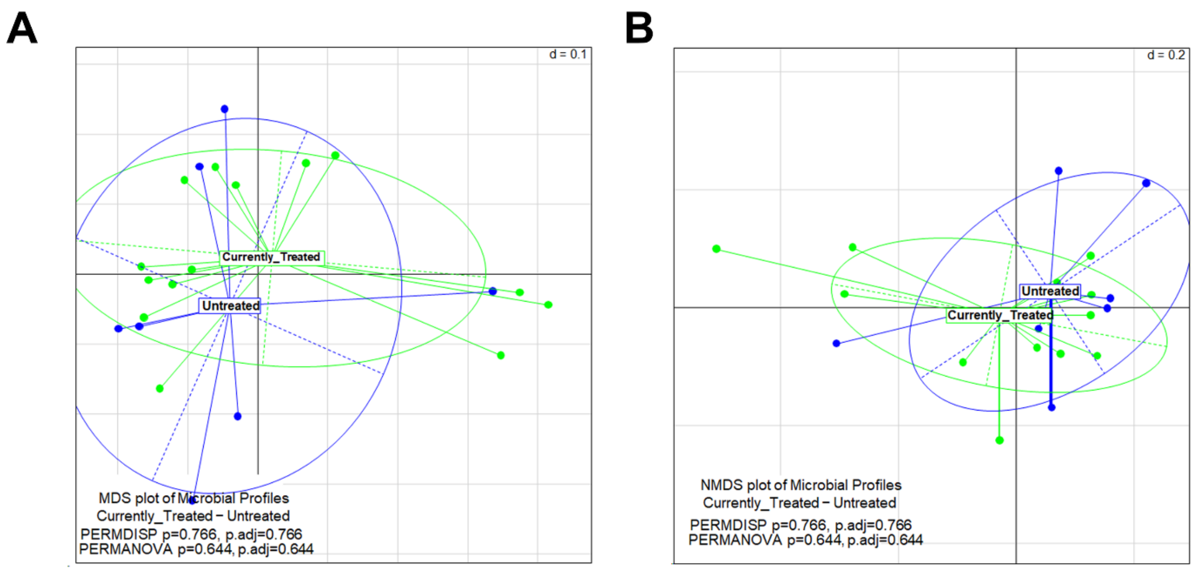

Supplementary Figure 3. Gut microbiota composition based on the use of biologics treatment. A MDS plot, **B** NMDS plot. No significant differences were observed between groups based on biologics treatment in the CD patient cohort (PERMDISP, p = 0.766; PERMANOVA, p = 0.644).

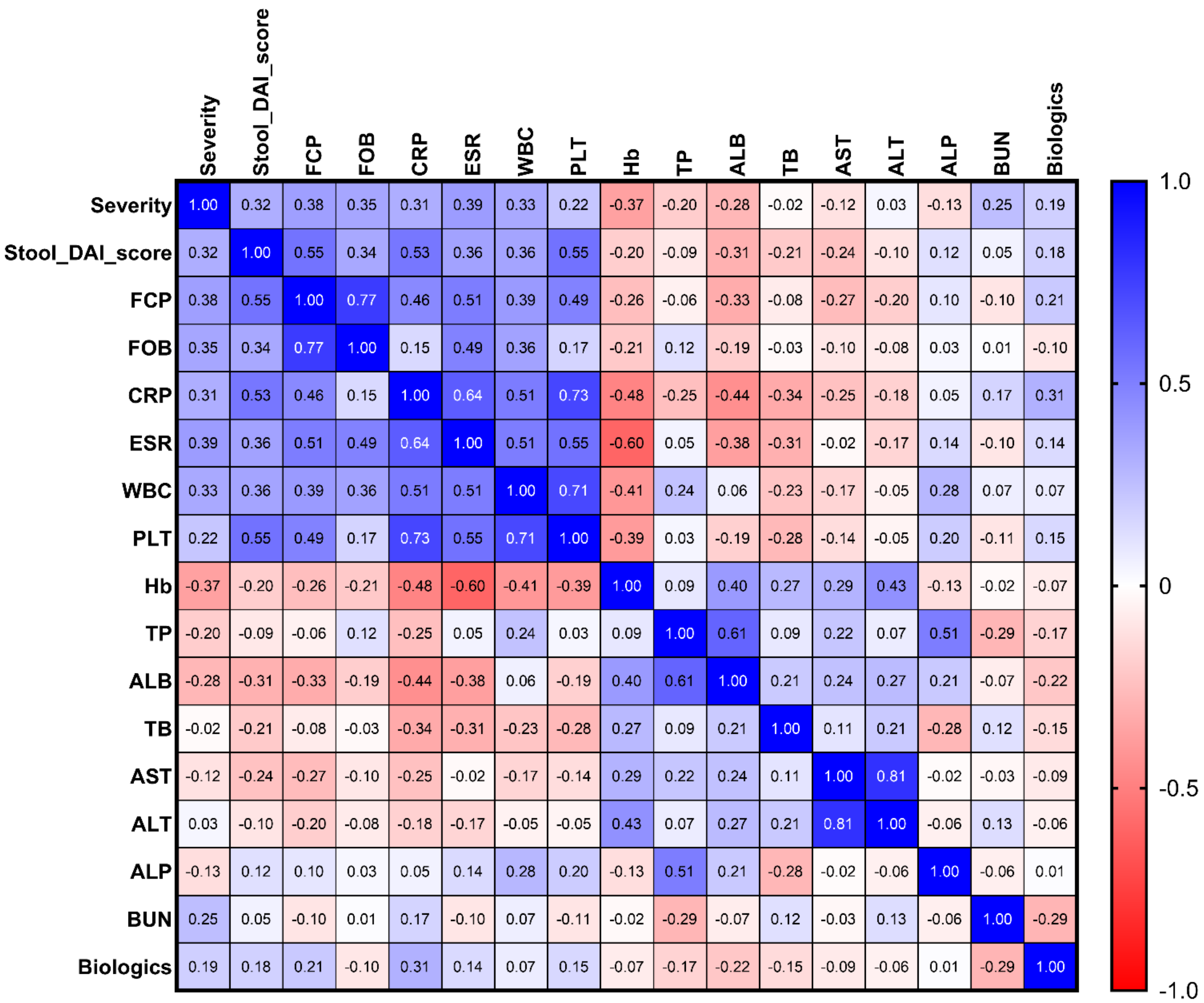

Supplementary Figure 4. Correlation between clinical indicator values in the IBD cohorts. The correlation was calculated using Pearson's correlation. FCP, Fecal calprotectin; FOB, Fecal occult blood; CRP, C-reactive protein; ESR, Erythrocyte sedimentation rate; WBC, White blood cells; PLT, Platelet; Hb, Hemoglobin; TP, Total protein; ALB, Albumin; TB, Total bilirubin; AST, Aspartate aminotransferase; ALT, Alanine aminotransferase; ALP, Alkaline phosphatase isoenzymes; BUN, Blood urea nitrogen.
