# Supplementary material for: Culture-based characterization of gut microbiota in inflammatory bowel disease
Source: Front Microbiol. 2025 Feb 20;16:1538620. doi: 10.3389/fmicb.2025.1538620 (PMC11884817; doi:10.3389/fmicb.2025.1538620)
Supplement: Supplementary file 1 [file Data_Sheet_1.ZIP › Supplementary_Materials/Supplementary_Tables.pdf]

Supplementary Tables

Supplementary Table 1. Differentially abundant taxa in gut microbiota between the UC and HC groups

No.	Taxa	Enriched group	LDA (log 10)	*p* value	*p*adj
1	*s__Collinsella_aerofaciens*	HC	4.3	< 0.001	< 0.001
2	*s__Alistipes_putredinis*	HC	3.9	0.026	0.026
3	*s__Parabacteroides_merdae*	HC	3.9	0.014	0.014
4	*s__Ruminococcus_callidus*	HC	3.7	0.008	0.008
5	*g__[Eubacterium]_ruminantium_group*	HC	3.7	0.025	0.025
6	*s__human_intestinal*	HC	3.6	0.011	0.011
7	*s__Prevotella_sp.*	HC	3.4	0.006	0.006
8	*s__Odoribacter_splanchnicus*	HC	3.4	0.006	0.006
9	*g__Tyzzerella_s__unidentified*	HC	3.3	0.022	0.022
10	*s__uncultured_Barnesiella*	HC	3.2	0.045	0.045
11	*s__Bacteroides_cellulosilyticus*	HC	3.2	0.027	0.027
12	*s__Burkholderia_sp.*	HC	3.1	0.024	0.024
13	*s__uncultured_Pasteurellaceae*	UC	3.8	0.041	0.041
14	*s__uncultured_Peptostreptococcaceae*	UC	3.6	0.007	0.007
15	*g__Sellimonas_s__uncultured_bacterium*	UC	3.6	0.032	0.032
16	*g__Lachnospiraceae_UCG-004*	UC	3.3	0.037	0.037
17	*s__[Clostridium]_spiroforme*	UC	3.2	0.048	0.048
18	*s__Bifidobacterium_dentium*	UC	3.1	0.015	0.015
19	*s__Butyricicoccus_sp.*	UC	3.1	0.041	0.041

Supplementary Table 2. Differentially abundant taxa in gut microbiota between the CD and HC groups

No.	Taxa	Enriched group	LDA (log 10)	*p* value	*p*adj
1	*s__uncultured_Enterobacterales*	CD	4.8	0.001	0.001
2	*s__uncultured_[Ruminococcus]_gnavus_group*	CD	4.6	0.001	0.001
3	*s__Erysipelatoclostridium_ramosum*	CD	4.2	0.027	0.027
4	*s__Faecalimonas_umbilicata*	CD	4.1	0.009	0.009
5	*s__uncultured_Lachnoclostridium*	CD	4.0	0.015	0.015
6	*s__uncultured_[Clostridium]_innocuum_group*	CD	3.9	0.004	0.004
7	*s__bacterium_NLAE-zl-G490*	CD	3.7	0.025	0.025
8	*s__uncultured_Alistipes*	CD	3.6	0.003	0.003
9	*s__Parasutterella_excrementihominis*	CD	3.5	0.049	0.049
10	*s__Odoribacter_splanchnicus*	CD	3.3	0.021	0.021
11	*g__Bifidobacterium*	HC	4.6	0.013	0.013
12	*s__Bacteroides_coprocola*	HC	4.5	< 0.001	< 0.001
13	*s__Bacteroides_uniformis*	HC	4.4	0.02	0.02
14	*s__Collinsella_aerofaciens*	HC	4.2	0.01	0.01
15	*s__uncultured_Bifidobacterium*	HC	4.1	0.016	0.016
16	*s__Parabacteroides_merdae*	HC	4.0	0.001	0.001
17	*s__Alistipes_putredinis*	HC	4.0	< 0.001	< 0.001
18	*s__uncultured_Agathobacter*	HC	4.0	0.008	0.008
19	*s__Ruminococcus_bicirculans*	HC	3.9	0.007	0.007
20	*s__human_intestinal*	HC	3.8	0.01	0.01
21	*s__Gemmiger_formicilis*	HC	3.8	0.043	0.043
22	*s__Ruminococcus_callidus*	HC	3.8	< 0.001	< 0.001
23	*s__Roseburia_inulinivorans*	HC	3.8	0.014	0.014
24	*g__Clostridia_UCG-014*	HC	3.7	0.036	0.036
25	*g__[Eubacterium]_ruminantium_group*	HC	3.7	0.001	0.001
26	*g__Subdoligranulum*	HC	3.7	0.005	0.005
27	*g__Alloprevotella_s__Prevotellaceae_bacterium*	HC	3.6	0.042	0.042
28	*g__Prevotella_9*	HC	3.6	0.042	0.042
29	*g__Lachnospiraceae_NK4A136_group*	HC	3.6	0.011	0.011
30	*s__Megasphaera_elsdenii*	HC	3.6	0.042	0.042
31	*g__Paraprevotella*	HC	3.6	0.005	0.005
32	*s__uncultured_Lachnospiraceae_ND3007_group*	HC	3.6	0.039	0.039

33	*s__Coprococcus_comes*	HC	3.5	0.018	0.018
34	*s__Paraprevotella_clara*	HC	3.5	0.004	0.004
35	*s__Alistipes_finegoldii*	HC	3.5	0.004	0.004
36	*s__Bifidobacterium_bifidum*	HC	3.5	0.029	0.029
37	*s__uncultured_Blautia*	HC	3.5	0.035	0.035
38	*s__Blautia_faecis*	HC	3.5	0.017	0.017
39	*g__Coprococcus*	HC	3.5	< 0.001	< 0.001
40	*g__UCG-002*	HC	3.4	0.018	0.018
41	*s__Roseburia_hominis*	HC	3.4	0.013	0.013
42	*s__Bacteroides_cellulosilyticus*	HC	3.4	< 0.001	< 0.001
43	*s__Prevotella_sp.*	HC	3.4	0.003	0.003
44	*g__[Eubacterium]_coprostanoligenes_group*	HC	3.3	0.006	0.006
45	*s__uncultured_UCG-002*	HC	3.3	0.042	0.042
46	*g__[Eubacterium]_ventriosum_group*	HC	3.3	0.024	0.024
47	*s__uncultured_Clostridiales*	HC	3.3	0.032	0.032
48	*g__NK4A214_group*	HC	3.2	0.015	0.015
49	*s__uncultured_Spirochaetales*	HC	3.2	0.042	0.042
50	*g__Subdoligranulum*	HC	3.2	0.033	0.033
51	*g__Lachnospiraceae_FCS020_group*	HC	3.1	0.021	0.021
52	*s__uncultured_UCG-005*	HC	3.1	0.001	0.001
53	*s__uncultured_UCG-003*	HC	3.1	0.001	0.001
54	*s__[Clostridium]_leptum*	HC	3.1	0.016	0.016
55	*s__uncultured_Acetivibrio*	HC	3.0	0.012	0.012

Supplementary Table 3. Clinical characteristics of cultured samples.

	HC	UC	CD	UC vs. HC (*P* value)	CD vs. HC (*P* value)	CD vs. UC (*P* value)
No. of subjects	8	10	10			
Age[a]	36.3 ± 2.3	53.0 ± 5.1	32.1 ± 2.2	**0.04**	0.15	**0.005**
Female, *n* (%)[b]	3 (38%)	7 (70%)	2 (20%)	0.34	0.61	0.07
Severity, *n* (%)[b]						
Mild	-	7 (70%)	6 (60%)	-	-	> 0.99
Moderate to severe	-	3 (30%)	4 (40%)	-	-	> 0.99
Biologic treatment, *n* (%)[b]						
Naïve	-	10 (100%)	2 (20%)	-	-	**0.0007**
Continuing	-	0 (0%)	7 (70%)	-	-	**0.003**
Stopped	-	0 (0%)	1 (10%)	-	-	> 0.99
ESR (mm/h)[a]	-	21.4 ± 6.7	30.9 ± 11.8	-	-	0.81
CRP (mg/dL)[a]	-	0.5 ± 0.4	2.4 ± 1.3	-	-	**0.008**
Stool characteristics, bacterial quantification, and inflammatory biomarkers						
Bristol scale 6 or 7, *n* (%)[b]	0 (0%)	1 (10%)	5 (50%)	> 0.99	**0.04**	0.14
Stool DAI[c]	0.1 ± 0.1	1.3 ± 0.5	2.6 ± 0.7	0.08	**0.002**	0.18
Log(bacteria cells/g stool)[c]	11.11 ± 0.23	10.56 ± 0.17	10.48 ± 0.14	0.06	**0.03**	0.76
FCP (mg/kg stool)[c]	56.2 ± 13.3	260.8 ± 100.6	973.9 ± 259.2	0.13	**0.002**	0.08
FOB (μg/kg stool)[c]	0.9 ± 0.9	35.5 ± 16.5	29.9 ± 10.8	**0.01**	**0.02**	0.77

Values are presented as number (%) or mean±SEM.

[a]Two-tailed Mann–Whitney test; [b]Two-sided Fisher's exact test; [c]One-way ANOVA using Kruskal–Wallis test.

ESR, Erythrocyte sedimentation rate; CRP, C-reactive protein in blood; FCP, Fecal calprotectin; FOB, Fecal occult blood; Stool DAI, Stool disease activity index.

Note. HC data was retrieved from our previous study (Park et al., 2024).

Supplementary Table 4. Putative novel species isolated from the gut microbiota in a culturomics study.

Name of species and strain	Novelty	Host	GenBank accession no.		Culture collection no.	Morphological characteristics
			16S rRNA	Draft genome		
Pseudogracilibacillus sp. ICA-222130	sp. nov.	UC	OP753731	SRR28033818	KCTC 43474, JCM 36064	long rods (0.5 × 1.4 – 4.5 µm); colonies were white, irregular, raised, and opaque.
Phocaeicola sp. ICN-14070	sp. nov.	UC	OP753733	SRR28033817	KCCM 90503, KCTC 25609, JCM 36144	cocci (0.8–0.9 µm in diameter); colonies were creamy white, convex, and circular ranging from 1 mm to 1.5 mm.
Selenobaculum gibii ICN-92133^{T}	gen. nov., sp. nov.	CD	ON753764	SRR23852394	KCTC 25622, JCM 36070	rod-shaped with a rounded end (0.4–0.5 × 1.6–3.2 µm); colonies were creamy white, convex, and circular forms less than 1 mm in diameter.

KCTC, Korean Collection for Type Cultures; JCM, Japan Collection of Microorganisms; KCCM, Korean Culture Center of Microorganisms. The novelty of *Selenobaculum gibii* strain ICN-92133^{T} was validated in a previous paper (doi:10.1038/s41598-023-42017-0).
